# Task integration and anticipation in complex, continuous motor tasks

**DOI:** 10.3389/fpsyg.2025.1557618

**Published:** 2025-06-09

**Authors:** Patrick Beißel, Stefan Künzell

**Affiliations:** Institute of Sports Sciences, University of Augsburg, Augsburg, Germany

**Keywords:** task integration, sequence learning, SRT task, anticipation, complex motor task, implicit motor learning

## Abstract

Multitasking and sequential motor learning research has advanced greatly in recent years, yet commonly accepted insights are largely based on simple, distinct tasks which cannot accurately reflect the variety of more complex and continuous tasks we encounter in everyday life. This study therefore aims to reassess the influence of task integration on motor sequence learning in complex, continuous tasks through the use of a virtual reality environment and an adapted SRT dual task suited for continuous movements. In our experiment, participants performed a complex, bimanual motor sequence task with varying degrees of suitability for task integration. We could successfully show that task integration has beneficial effects on complex task acquisition if covariations between tasks are consistent and detrimental effects if covariations are too inconsitent or missing. Minor inconsistencies within a repeated sequence can however be mitigated. These results highlight the distinct influence of task integration on complex, continuous motor learning, yet emphasize the need for further research beyond distinct, simple tasks.

## 1 Introduction

Sequence learning is a research area of undiminished relevancy, as motor sequences frequently represent the basis for routinely performed or highly efficient tasks, from recreationally playing the piano or driving a car to competing with the world’s best athletes in professional sports. An interesting example for the latter can be found in speed climbing, which is one of three formats of sports climbing introduced during the 2020 Summer Olympics in Tokyo, Japan. Speed climbing can be described as a head-to-head vertical sprint up a standardized 15-meter wall (International Federation of Sport Climbing, 2022; Nguyen et al., 2022) and represents a complex, continuous motor task which requires coordination of multiple effector-specific motor sequences. Task integration, the ability to (implicitly) perceive and learn two or more distinct tasks as one (Beißel and Künzell, 2024; Künzell et al., 2018; Schmidtke and Heuer, 1997), is bound to distinctively influence this skill. In order to compete with other climbers in this discipline, athletes first must identify, learn and integrate the individual set of sequences best suited to their respective anthropometric characteristics, power and strength (Winkler et al., 2023). The learning process of such complex motor tasks is of great interest to scientists and coaches alike, but motor learning research in the past has largely focused on simple motor tasks with discrete trials (Haar et al., 2021; Lee and Anderson, 2001; Levac et al., 2019; Wulf and Shea, 2002; also Pelzer et al., 2022b; Röttger et al., 2021). These discrete trials usually omit anticipatory preplanning of subsequent trials parallel to motor execution (Ariani and Diedrichsen, 2019; Dahm and Krause, 2024) which is a feature and possible benefit of many everyday tasks. This is naturally owed to better experimental control of simple tasks in the laboratory and the challenges of validly assessing more complex or continuous tasks, yet more research on complex skills is certainly needed (Haar et al., 2021; Levac et al., 2019; Wulf and Shea, 2002). However, insights about motor learning gleaned from simple task studies cannot directly be transferred to complex tasks or continuous tasks allowing for anticipation of subsequent motor execution decisions (Lee and Anderson, 2001; Levac et al., 2019; Sternad et al., 2014; Wulf and Shea, 2002). We therefore identify three research gaps in contemporary motor learning research that need to be expanded upon: the influence of task integration on motor sequence learning is commonly accepted, yet details about its extent and robustness against disturbance remain largely unexplored. And as research in this field has widely focused on simple tasks with discrete trials, research needs to be expanded to both complex tasks, as well as continuous tasks that allow for anticipation of subsequent actions. Expanding research into these areas requires different experimental settings for validly researching motor skills, which recent advances in virtual reality (VR) technology might provide.

Despite the necessity for closer examination of task integration, past research has provided fundamental knowledge of its core aspects. It is currently understood as the implicit process of perceiving and then merging two or more distinct tasks into one gestalt during task acquisition (Klapp and Jagacinski, 2011; Künzell et al., 2018). Tasks and entire sequences are thus treated as functional, inseparable units (Schmidtke and Heuer, 1997). Depending on the structure of the tasks, task integration can have a beneficial or detrimental influence on the learning process, as it is a perpetually active process (Pelzer et al., 2022a; Pelzer et al., 2022b; Röttger et al., 2021; Zhao et al., 2020). The introduction of secondary tasks alongside a primary task usually leads to dual-task interference and a subsequent decline in performance due to competition for limited mental resources, a limited central capacity, or perturbations resulting from increased demands for judgement and decision-making (Broeker et al., 2018; Koch et al., 2018; Schmidtke and Heuer, 1997). Task integration can mitigate these detrimental effects if repeating covariations between the tasks can be identified and integrated into a joint memory episode through associative chaining, leading to improved learning and performance (Beißel and Künzell, 2024; de Oliveira et al., 2017; Pelzer et al., 2022b; Röttger et al., 2021; Schmidtke and Heuer, 1997; Swinnen and Wenderoth, 2004). Conversely, task acquisition can be severely disrupted if no covariations exist between two tasks and integration is nonetheless attempted, leading to task confusion as the dissimilar, distinct tasks can neither be integrated, nor separated (Beißel and Künzell, 2024; Hazeltine and Schumacher, 2016; Röttger et al., 2021). Task confusion and resulting motor detriments can also arise from discrepancies between successfully learned, reactivated memory episodes and altered task demands (Frings et al., 2020; Pelzer et al., 2022b). We showed in a previous paper that this is by no means a binary distinction: task integration can still be effective in spite of partial random disturbances in the secondary task, as the cognitive system is capable of freeing up resources by focusing on the structured parts of a task (Beißel and Künzell, 2024; Broeker et al., 2021). There is however a limit to the benefits of this mitigating capacity, as comparatively small amounts of integrable items in an otherwise mostly random sequence seem to be detected, but unsuccessful integration attempts lead to excessive resource expenditure (Beißel and Künzell, 2024). Yet this does not fully suppress other learning mechanisms as some effector-specific learning still takes place. We may summarize that successful task integration has beneficial effects on task acquisition and performance when covariations are consistent enough to be identified and integrated, while the effect is conversely detrimental when these conditions are not met (Beißel and Künzell, 2024; de Oliveira et al., 2017; Röttger et al., 2021; Schmidtke and Heuer, 1997). Yet the exact processes behind task integration are still not fully researched and perspectives on it have changed since the groundwork laid by Schmidtke and Heuer (1997). It is necessary to study the impact of task integration under more ecologically valid circumstances, such as in complex, continuous and predictable tasks which might be inherently detrimental to integration processes.

Task integration might impact more realistic tasks differently, as they often require continuous sensorimotor and cognitive attention (Johannsen et al., 2022). Most daily tasks also do not suddenly appear before being performed but can be anticipated and thus subsequent actions can be planned ahead (Ariani and Diedrichsen, 2019; Dahm and Krause, 2024). Such anticipatory cues might even hurt sequence learning and automatization, as a purely reactive mode without reactivation of motor memory episodes could be adopted (Dahm and Krause, 2024). In this context, Ariani and Diedrichsen (2019) distinguish between preplanning and online planning. Preplanning can be utilized when sequence information is provided before movement is initiated but is unlikely to be effective when sequences are too long, or preparation time is too short (Ariani and Diedrichsen, 2019; Dahm and Krause, 2024; Haith et al., 2016). Online planning refers to the selection of subsequent actions during the execution of current actions and thus allows for the successful handling of successive, rapid stimuli presentations, as playing the piano or speed climbing would entail (Ariani and Diedrichsen, 2019; Dahm and Krause, 2024). While previous studies have shown that anticipatory cues and concomitant planning can reduce dual-task costs, the effects of prediction on task integration and sequence learning remain unclear, especially for complex tasks (Broeker et al., 2017; Dahm and Krause, 2024).

Complex tasks might be impacted by task integration and other motor learning mechanisms differently than simple ones due to increased resource demands. Learners faced with a rather simple task will form a rudimentary action plan which will then be adapted and built upon in subsequent interactions until it is adequate for the task (Guadagnoli and Lee, 2004). When confronted with a more complex skill with more degrees of freedom or multiple viable solutions, however, learners might be forced to focus on parts of the new skill and as such parts of the developing movement representation might at first be divided into relatively independent subcomponents which can then be learned easier and founded on more basic learning principles (Guadagnoli and Lee, 2004; Lee and Anderson, 2001). Lee and Anderson (2001) call this the reducibility hypothesis. This approach would consequently require more mental and physical resources compared to simpler tasks, as has been shown in previous studies. Meister et al. (2005) reported higher fMRI activations of the pre-supplementary motor area and the rostral part of the premotor cortex for complex sequences performed on a keyboard in contrast to simple sequences. In a more recent study, Mussini et al. (2021) compared motor and cognitive preparatory brain activity during simple and complex visual motor discriminative response tasks and reported less neural activity in simple tasks in comparison to complex tasks. These and further studies (e.g., Deroost and Soetens, 2006; Jarus and Gutman, 2001) support the notion that research results from simple tasks cannot directly be transferred to complex tasks (Lee and Anderson, 2001; Levac et al., 2019; Sternad et al., 2014; Wulf and Shea, 2002), especially as simple motor skills, such as pressing buttons in response to distinct stimuli, are either learned quickly or already perfected. Complex skills, usually used in response to more complex or numerous stimuli, offer a wider solution space and thus require a greater degree of adaption (Levac et al., 2019; Sternad et al., 2014). Yet most previous studies do not explicitly define what constitutes a complex task and mention defining features such as higher time demands, higher rates of errors, more difficult sequences, greater practice requirements or cognitive demands (Du and Clark, 2018; Holper et al., 2009; Levac et al., 2019; Verstynen et al., 2005; also Deroost and Soetens, 2006; Gajewski and Falkenstein, 2013; Jarus and Gutman, 2001). We agree with Levac et al. (2019) definition of a complex task as having nested redundancy, meaning that next to having more execution variables than task defining variables, complex tasks also have intrinsic, extrinsic and task redundancy. Building upon the ground laying work of Bernstein (1967), these three aspects of redundancy describe the infinitely different configurations of effector joint angles, trajectories to the target and acceptable target locations to successfully complete a given task. This high degree of redundancy in turn introduces variability into complex tasks which represents exploration within the new task space that can adapt new motor responses or connect to existing, better suited ones by extracting communalities and differences (Cardis et al., 2018; Dhawale et al., 2017; Hossner and Zahno, 2022; Latash, 2012). This intentional variability and exploration might inadvertently counteract task integration processes, which normally attempt to reduce environmental complexity through integrating covariations.

Based on these insights we argue that complex and predictable tasks impose conditions on motor learning that may diverge from prior findings derived from simple, discrete tasks. More studies on motor learning of complex tasks with nested redundancy are thus needed. There have been studies which have advanced the research in this area: Du and Clark (2018) have adapted the serial reaction time (SRT) task to study implicit motor sequence learning with a foot-stepping task and could show successful sequence acquisition in several experiments (Du and Clark, 2017; Du et al., 2017). Similarly, Baird and Stewart (2018) used stereoscopic glasses, a projector, and an electromagnetic marker on participants’ index fingers in a whole-arm, three dimensional reaching task in a virtual environment. Kinematic data of participants reaching for nine different target spheres which appeared in either a repeated or random order revealed successful sequence learning in this complex task. Also, Sense and van Rijn (2018) adapted the classic SRT task to VR and had participants wearing a VR headset reach for four different target locations with their dominant hand. The target locations lit up following a probabilistic motor sequence, while response time and error rates were used as dependent variables. The results were comparable to traditional SRT experiments, thus validating VR as a viable research tool for motor learning. We believe that complex motor research can greatly benefit from using VR setups, as it is a viable and promising research tool, which is also a cost-effective, flexible and accessible approach to assessing complex tasks (Levac et al., 2019). In our own previous study, we could successfully show that task integration does affect the learning of complex, bimanual dual tasks positively if the underlying sequence structures are sufficiently compatible, and negatively if they are not. It appears to be a dominant learning mechanism that is also to some degree resistant against random perturbations disrupting sequence acquisition. Yet while we could provide these novel insights into complex motor sequence learning, the experimental VR setup we utilized was comprised of distinctly separated trials without the possibility of anticipating upcoming trials beyond the learning of the underlying sequence. As this approach was still far removed from many motor tasks performed outside the laboratory, we aim to re-evaluate our findings in the context of complex, continuous motor tasks that allow for predictive online planning. We utilized a VR environment to implement an adaptation of the SRT task incorporating a complex, bimanual dual task. Past studies have shown that bimanual tasks are a form of multitasking faced with performance limitations, increased complexity, and a reduction of task stability and accuracy, especially if the tasks assigned to each limb differ in regard to task execution and/or are performed simultaneously (Beißel and Künzell, 2024; Hazeltine et al., 2006; Swinnen and Wenderoth, 2004; Wenderoth et al., 2002). We therefore used errors, accuracy and time as dependent variables. The dual task required continuous movement and allowed for predictive online planning to address previously mentioned research gaps. It also featured two parallel, underlying motor sequences. These sequences exhibited varying degrees of compatibility for integration, depending on our five groups, ranging from ideal to impossible for task integration. Additionally, an implicit learning score (ILS) was calculated for each dependent variable from the difference between performance in then familiar trials compared to a random catch trial (Beißel and Künzell, 2024; Rubino et al., 2025; Schmidtke and Heuer, 1997). The ILS reflects the implicit learning gain derived from acquiring the motor sequences, compared to purely use-dependent learning (Wolpert et al., 2011). With this methodological approach, ensuring a complex, continuous, and predictable motor task, we tested two hypotheses. First, task integration affects performance and learning with more beneficial effects for more compatible sequence structures and more detrimental effects for less compatible sequence structures. This should mirror the results of studies on simple, discrete motor tasks and includes the assumption that motor improvement can be attributed to sequence learning beyond use-dependent learning. Second, we assume that performance improvement through task integration can occur even if it is impaired by random sub-sequences, as long as the integrable, regular sub-sequences are long enough. Both hypotheses will be mainly assessed based on differences between groups. As a secondary goal, for future use, we aim to assess the validity of our methodological approach for assessing complex, continuous tasks with an adapted SRT task.

## 2 Materials and methods

### 2.1 Participants

One hundred participants took part in this experiment. Only self-reported, right-handed people between the age of 18 years and 30 years were accepted into the experiment. The participants’ mean age was 23.65 (SD = 3.49) years, while 53 were female and 47 male. They were assigned to one of five groups (*n* = 20) based on their performance in the familiarization block (see below). As in our prior experiment (Beißel and Künzell, 2024), group size was determined through a G-Power (Faul et al., 2007) *a priori* sample size calculation based on the implicit-learning score analysis by Schmidtke and Heuer (1997). For a one-way ANOVA with five groups, a main effect of group of *f* = 0.56, and an *α* error probability of 0.05, the power analysis (1 − *β* = 0.95) indicated a minimum of 13 participants per group (*N* = 65). Group size was increased to 20 to reduce the chance of type II errors and increase statistical power (Lakens, 2022; McKay et al., 2023). Findings of this study should generally be transferable to healthy, non-elderly and non-learning-impaired adults.

### 2.2 Apparatus

Participants in this experiment wore a VR headset and controllers on both hands. The Valve Index VR headset contained two 1,440 × 1,600 LCD IPS fast switching displays with refresh rates of 144 Hz and a field of view of 135°. The Valve Index controllers were fastened to the hands and contained 87 sensors for measuring hand positions and applied pressure, as well as an accelerometer for measuring linear acceleration. Both were tracked by two stationary base stations with a Lighthouse 2.0 tracking system, positioned in opposite corners of a 6 m × 6 m area. While the latency between moving the controllers and the movement being displayed on this headset’s displays could be expected to be on average 28.7 ms for sudden movements and 12.1 ms for continuous movements (Warburton et al., 2023), we measured a maximal latency of 6.9 ms and average latencies of 3.6 ms. Values below 90 ms are generally considered to not affect participants’ performance (Kelkkanen et al., 2023). As a basis for stimulus presentation, we used the level editor of the rhythm game Beatsaber, published by Beat Games and programmed in Unity.

### 2.3 Design and procedure

**Figure 1:**
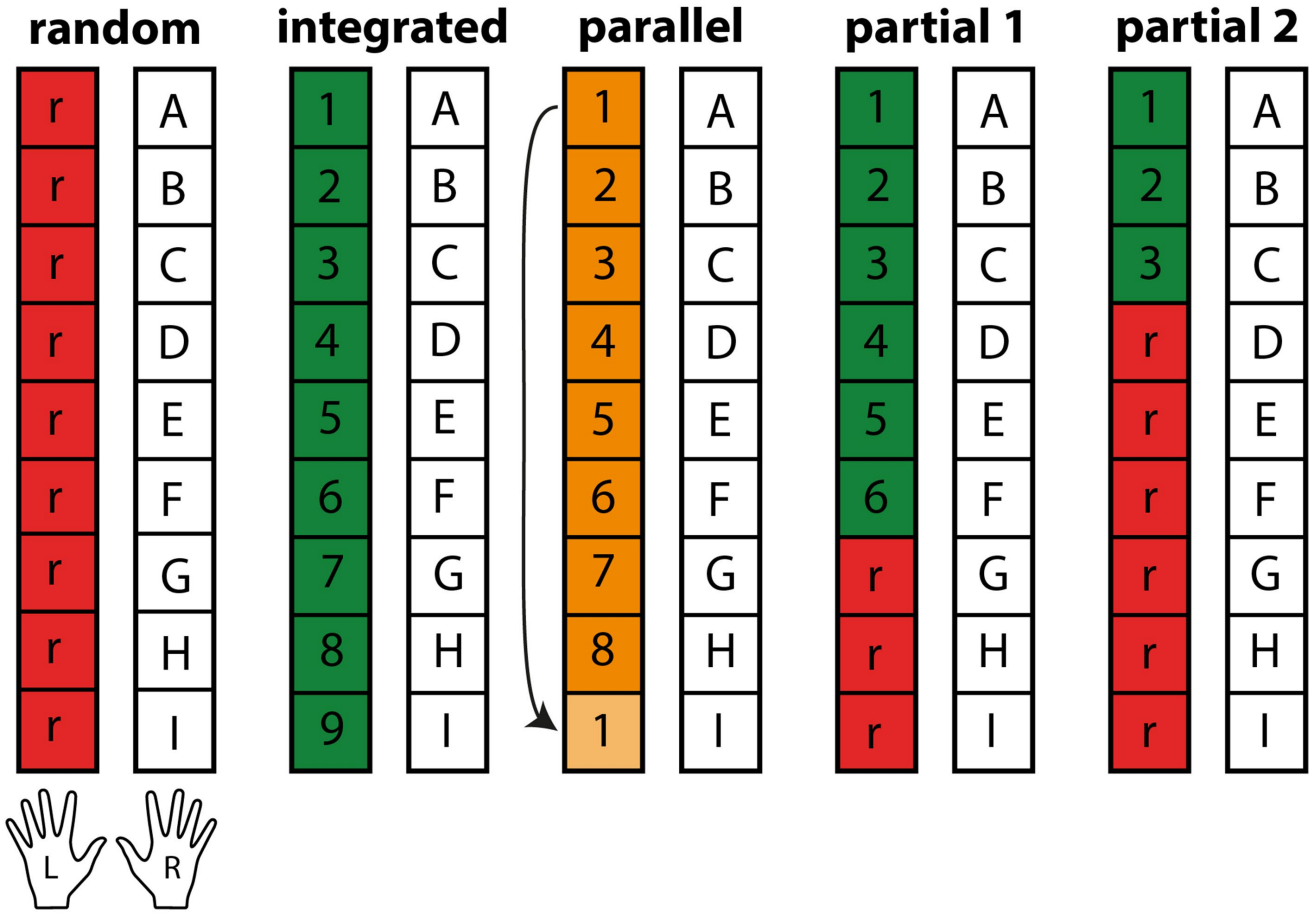
Task structure for dual-task groups (*n* = 20). The repeating right-hand sequence shared by all groups is represented by the letters “A–F,” numbers represent repeating sequence elements and “r” random stimuli. For each non-catch-trial block, the sequence was repeated eight times for a total of 72 trial pairs. The Parallel group continued the next sequence with the successor of the stimulus last completed, here with stimulus No. 2.

All participants performed a serial, bimanual dual task, using both arms simultaneously to interact with sequenced stimulus pairs appearing near them. The primary task was performed with the dominant, right hand, the secondary task with the left hand. Subsequent stimulus pairs beyond the most current one could be seen in advance. The groups were set apart by the respective sequence design of their secondary, left-hand task (see Figure 1). The groups will be referred to as the Random, Parallel, Integrated, Partial 1 and Partial 2 group. All groups had a fixed, repeating sequence of nine items on their right hand, which should be long enough to evade explicit awareness (Baird and Stewart, 2018; Nissen and Bullemer, 1987; Robertson, 2007; Sense and van Rijn, 2018). The Random control group had a random sequence on their left hand, making any attempts at successful task integration impossible. The Parallel group had a repeating, parallel sequence of eight items on their left hand, allowing for effector-specific sequence learning, but not for task integration. The Integrated group had ideal conditions for task integration with nine-item sequences on each of their hands. The partially sequenced groups Partial 1 and Partial 2 could potentially integrate parts of their left-hand sequence, with a distribution of six to three and three to six sequenced and random parts, to allow for assessment of partial sequence learning in the presence of random interferences. Based on our prior experiment, we expected the Integrated group to exhibit the greatest learning improvements, followed in descending order by the Partial 1, Parallel, Random and Partial 2 group. This order reflects our hypotheses: First, the Integrated group should benefit most from task integration due to its ideal sequence compatibility compared to the three groups mentioned last. Second, despite the short random sub-sequence, the Partial 1 group should be able to integrate the two tasks and therefore perform similarly well compared to the Integrated group, yet significantly better than the Parallel, Random and Partial 2 group. We did not expect major learning differences between the latter three groups as their potential for successful task integration is small or non-existent, but they still serve important purposes. If the Integrated and Partial 1 group surpass the others, we can assume that successful task integration facilitates learning of the underlying sequence and that minor disturbances can be mitigated. If Partial 2 were to improve more than Random and Parallel, it would indicate that even minor regularities within random interferences can be perceived and learned effectively. If the Parallel group were to perform well, it would indicate that alternative learning processes such as effector-specific learning are equally present and utilized, attenuating the importance of task integration in dual-tasks. The Random group, having the smallest chance for effective learning, serves as the control group.

**Figure 2:**
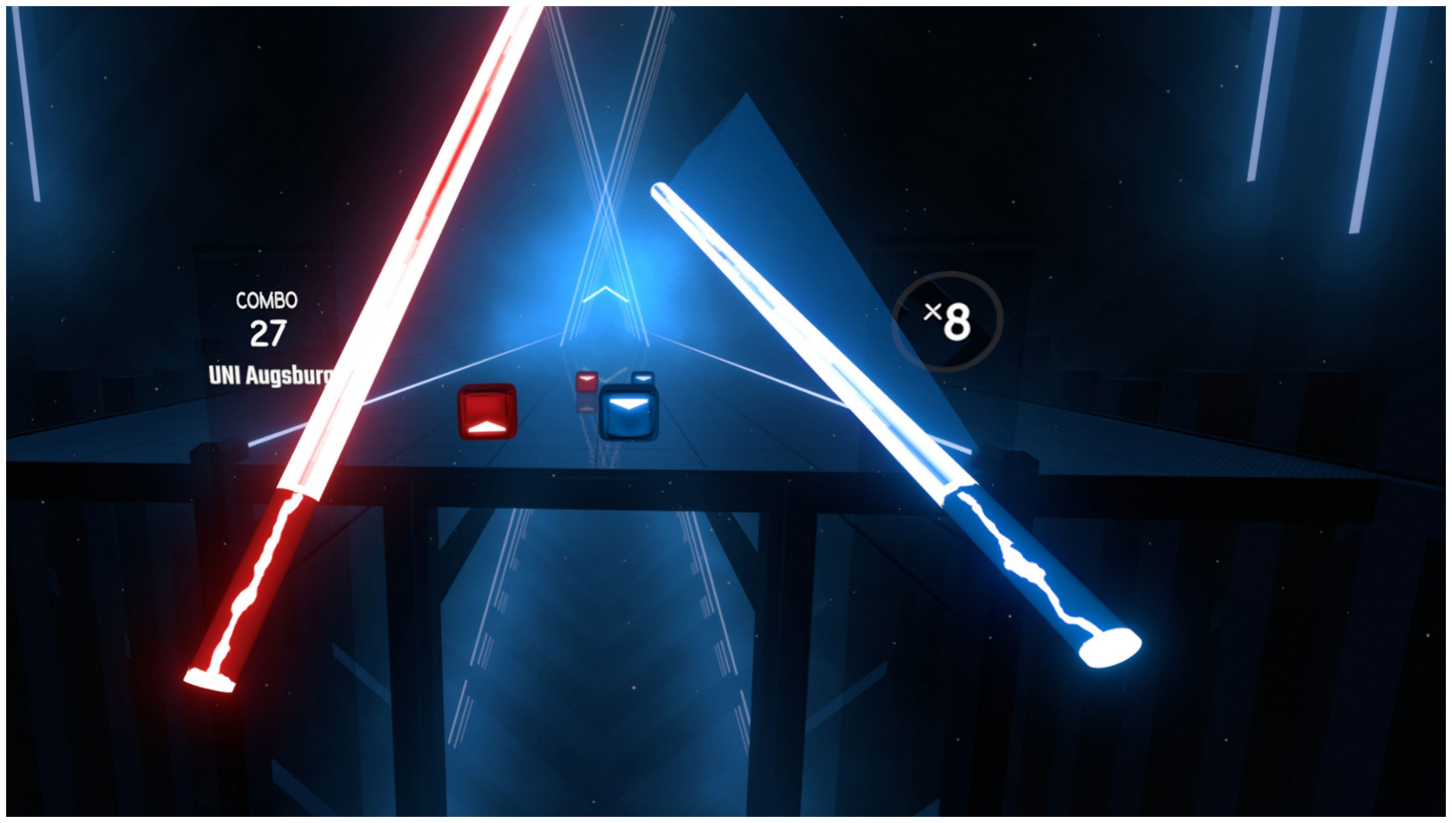
Main task as seen from participants’ perspective. Cubes appeared towards the far side of the indicated path before moving towards participants on a straight trajectory. A combo counter on the left, which added the number of hits without mistakes, and a points multiplier on the right served as feedback for participants’ performance.

At the start of the experiment, participants were informed that coordination and accuracy of movements in a bimanual task would be assessed. After moving to the center of the designated area, they were then instructed in the proper use of the VR headset and controllers. In VR, the participant stood on a virtual platform, with a path framed by lights leading towards it (see Figure 2). White footprints marked the ideal spot to stand on, participants were asked to remain on them. In their right and left virtual hands, respectively, they held a blue and red sword. The general task was hitting pairs of blue and red cubes with directional arrows on them which appeared some distance away from participants and then moved towards and ultimately past them. Participants were asked to hit the cubes in the indicated directions, to always aim for the center and, if possible, to use big, flowing movements for additional points. Successfully hit cubes disappeared immediately with a slashing sound. Pairs of stimuli appeared in a fixed 0.45 s rhythm, determined in pre-experiments, to ensure continuous movement without overwhelming participants. Participants could always see two pairs of stimuli during the experiment, allowing for anticipation beyond single trials. After a pair of cubes was hit or moved past the participant, the next pair of cubes appeared behind the remaining one. Stimuli appeared in an invisible, rectangular grid four columns wide and three rows high, with blue, right-handed cubes only appearing in the right half and vice versa. With a total of four possible hit directions in the horizontal and vertical axes, this resulted in 24 different stimuli per hand and a combined total of 576. Feedback for successful hits was given visually, auditorily and through vibration of the controller, as multimodal feedback can enhance complex motor task learning (Sigrist et al., 2015). Feedback to misses was given visually and auditorily.

After the first instruction, participants completed a familiarization block with 112 random stimulus pairs appearing rather slowly in the beginning and then accelerating to standard speed towards the end. This block was repeated three times and a predefined minimal score had to be reached to participate. The score reached in the third block was used to assign participants to their respective groups and to ensure group homogeneity. After group assignment, participants went through two practice phases and a test phase, separated from each other by five-minute breaks. All phases consisted of six blocks, each block with 144 stimulus pairs, and separated by 30-s breaks. The aforementioned nine-item sequence structure was repeated eight times and used throughout all blocks, with the exception of blocks three and four in the test phase, which were random and used as a catch trial to assess learning in a post-acquisition test (Müssgens and Ullén, 2015). The experiment concluded with a short interview to evaluate the degree of implicit/explicit learning involved, although current research has shown that a clear distinction between those categories is close to impossible in methodology and practice alike, especially for SRT tasks (Hadjiosif and Krakauer, 2021; Maresch et al., 2021; Moisello et al., 2009). The whole experiment lasted about 45 min.

The following dependent variables were measured during the experiment:

- Correct Behavior (CB) [%], indicating whether both cubes of a pair were hit from the correct direction and with the correct sword. CB is represented by percentage of correct hits to total trials, with 100% indicating no mistakes. Correct Behavior should increase over time and be highest in the Integrated group, lowest in the Random group.
- Distance to Center (DtC) [%], serving as an indicator for accuracy by measuring the distance between hit trajectory and cube center, with a perfectly centered hit showing 0% deviation and increasing percentages towards the edges of the cube. Accuracy should increase with practice (Moisello et al., 2009), leading to a reduction of percentages.
- Time Deviation (TD) [ms], measuring the deviations from hitting the cubes in the ideal, predefined rhythm of 0.45 s in a spot within comfortable reach of the participant. Greater Time Deviation should indicate stress or hesitation, resulting in overreach or belated hits, while a reduction in Time Deviation should indicate successful learning.

Furthermore, the ILS was calculated for each variable and reflects the respective learning gain (see *Data Processing and Analysis*), which should be highest in the Integrated group, lower for the other groups as indicated earlier. Further data was collected during the experiment but not intended as main variables: effector-specific Correct Behavior, a multiplier increasing points with a factor of 2, 4 or 8 corresponding to 2/4/8 hits without mistakes and a point score composed of DtC, pre- and post-hit swing angle.

### 2.4 Data processing and analysis

Data and programs mentioned here can be accessed online.^1^ Data used for dependent variables were extracted from Beatsaber, which records the controllers’ positional data as well as interaction with cubes, including hits, misses, and accuracy through point of contact with individual cubes. A unity-based plug-in using ModAssistant extracted data during the experiment and recorded it in individual .csv-files, one for each block of cubes. The data from these files was aggregated into one file containing the variables for all blocks and participants. CB was calculated from percentage of correct hits to total trials. For DtC and TD, means were used to summarize the participants’ performance in each block. Incorrect behavior was excluded for these variables so as to avoid data inflation or dilution through outliers, which should improve the signal-to-noise ratio and help with focusing on learning effects (Berger and Kiefer, 2021; Vankov, 2023). Also, errors are recorded through CB. To measure and compare learning effects, the ILS was calculated for each variable during the test phase, measuring the mean difference between the familiar blocks two and five and the random blocks three and four. Data was then analyzed using Jamovi, version 1.6.23 (The Jamovi Project, 2021). Shapiro–Wilk tests were used to check data sets for normality distribution and parametric or non-parametric tests were consequently used as indicated in results. Homogeneity of variance was tested for ANOVAs using Levene’s test and either Games-Howell or Tukey *post-hoc* tests were used accordingly.

### 2.5 Transparency and openness

We report how we determined our sample size, all data exclusions (none excluded), all manipulations and all measures in the study. This study’s design and its analysis were not pre-registered. The study was approved by the committee for ethics of the University of Augsburg.

## 3 Results

Prior to statistical analysis, the interview was evaluated and a total of 10 of 100 participants with a degree of explicit awareness of the used sequence were found. These were spread over the groups, with none in the Random group, two each in the Parallel and Integrated groups, five in the Partial 1 group and one in the Partial 2 group. Performance improvements can therefore be cautiously attributed to implicit learning processes.

To ensure successful group assignment and the validity of between-group comparisons, we first verified no differences existed between groups with a baseline comparison of the last familiarization block using one-way between-groups analyses of variance (ANOVAs) with the dependent variables “CB” [%], “DtC” [%] and “TD” [ms], the grouping variable “group” No significant differences were found, *F* (4, 95) = 0.24/0.46/0.23; *p* = 0.914/0.763/0.921; *η*^2^_part_ = 0.01/0.02/0.01. Means for the individual variables ranged from 58.0 (SD = 8.6) to 60.8 (SD = 10.8) for CB, 24.0 (SD = 2.6) to 25.4 (SD = 4.6) for DtC, and 9.75 (SD = 10.7) to 12.5 (SD = 12.9) for TD.

**Figure 3:**
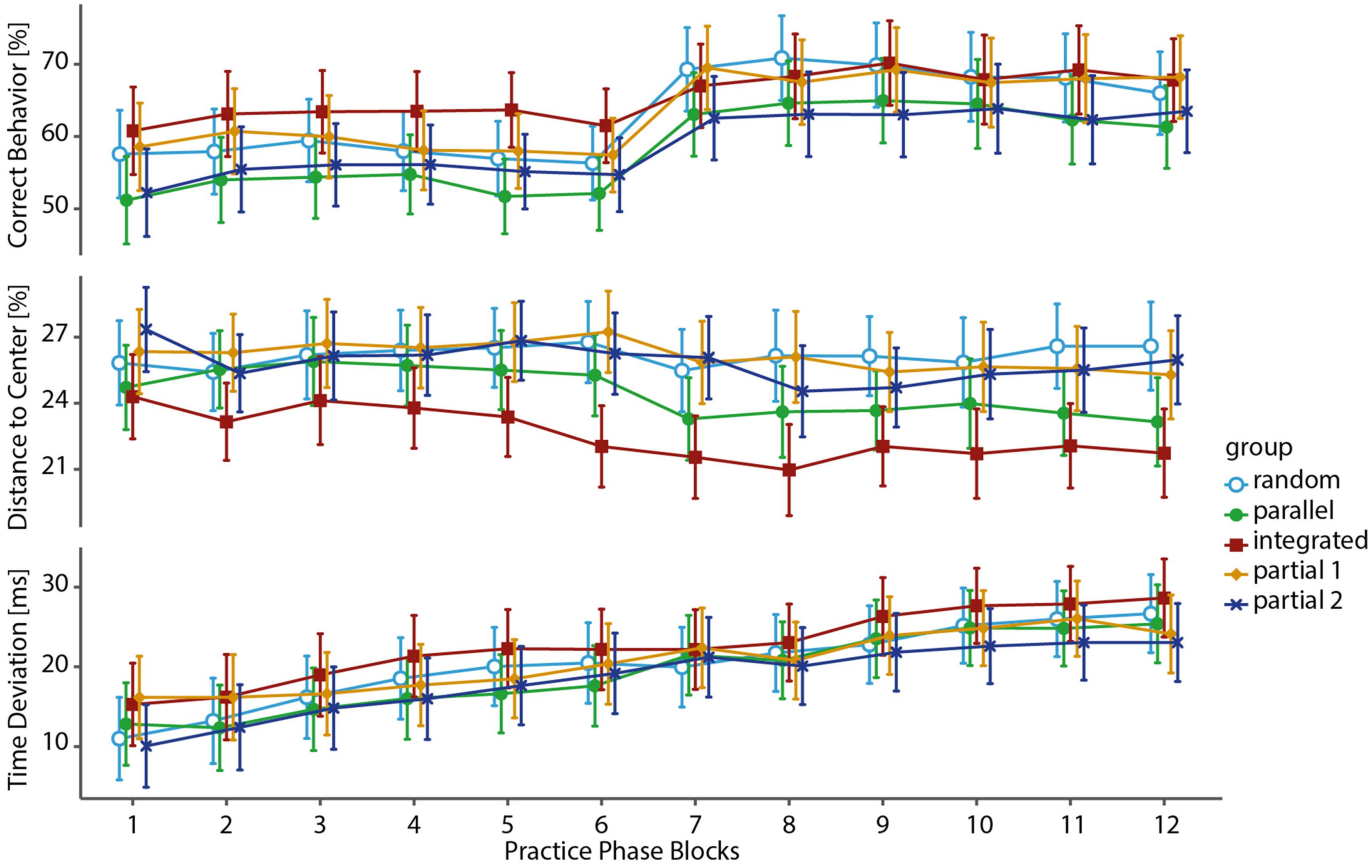
Performance changes during practice phase. The graphs show the means (95% CI) of each practice block for each variable and group. All groups significantly improved their performance over the course of the practice phase. CB improved notably after the break between blocks six and seven, while DtC and TD improved more gradually. No significant differences between groups were found.

As the next step, we assessed whether any performance increase and thus learning had taken place during the practice phase by comparing the first and last block in regards to group-specific differences and differences between groups with a mixed ANOVA for each variable with the within-subject factor “block” (1st block, 12th block) and the between-subject factor “group” (Random, Parallel, Integrated, Partial 1, Partial 2) (see Table 1 and Figure 3 for overview). While significant main effects for “block” could be shown for all groups and variables, a significant main effect for “group” could only be shown for DtC and no significant “block × group” interaction was found. Despite the lack of block by group interaction, we decided to follow up with Dunn–Bonferroni *post-hoc* comparisons focused on within-group differences, as these are not sufficiently reflected in the ANOVA’s *F*-statistic and differences might not be detected (Midway et al., 2020).

**Table 1:** Practice phase learning: mixed ANOVA.

Dependent variables	Main effect “block”	Main effect “group”	Main effect “block × group”
CB [%]	*F* (1, 95) = 94.4	*F* (4, 95) = 1.61	*F* (4, 95) = 0.583
	*p* < 0.001	*p* = 0.179	*p* = 0.676
	*η*^2^_part_ = 0.50	*η*^2^_part_ = 0.063	*η*^2^_part_ = 0.024
DtC [%]	*F* (1, 95) = 6.22	*F* (4, 95) = 3.51	*F* (4, 95) = 1.36
	*p* = 0.014	*p* = 0.010	*p* = 0.254
	*η*^2^_part_ = 0.061	*η*^2^_part_ = 0.129	*η*^2^_part_ = 0.054
TD [ms]	*F* (1, 95) = 177.83	*F* (4, 95) = 0.727	*F* (4, 95) = 1.8
	*p* < 0.001	*p* = 0.576	*p* = 0.141
	*η*^2^_part_ = 0.65	*η*^2^_part_ = 0.03	*η*^2^_part_ = 0.07

CB = Correct Behavior, DtC = Distance to Center, TD = Time Deviation. *F*-, *p*-values, and effect sizes for dependent variables CB, DtC and TD.

The results showed a significant within-group increase in Correct Behavior and Time Deviation over the practice phase for all groups. No significant changes in accuracy could be found for Distance to Center, although all groups’ means except for the Random group improved on their accuracy. In summary, clear evidence for within-group learning in two variables can be shown for the practice phase, yet no differences between groups could be found prior to the test phase (see Table 2).

**Table 2:** Practice phase within-group learning: *post-hoc* comparisons for all groups.

Group	Block	CB [%]	DtC [%]	TD [ms]
Random	Block 1 (SE)	57.6 (3.05)	25.8 (0.96)	11.0 (2.61)
	Block 12 (SE)	66.0 (2.89)	26.6 (1.01)	26.7 (2.46)
	*p*-value	0.006	0.999	<0.001
Parallel	Block 1 (SE)	51.2 (3.05)	24.7 (0.96)	12.8 (2.61)
	Block 12 (SE)	61.3 (2.89)	23.2 (1.01)	25.4 (2.46)
	*p*-value	<0.001	0.999	<0.001
Integrated	Block 1 (SE)	60.8 (3.05)	24.3 (0.96)	15.3 (2.61)
	Block 12 (SE)	67.8 (2.89)	21.7 (1.01)	28.6 (2.46)
	*p*-value	0.045	0.697	<0.001
Partial 1	Block 1 (SE)	58.6 (3.05)	26.3 (0.96)	16.2 (2.61)
	Block 12 (SE)	68.2 (2.89)	25.3 (1.01)	24.1 (2.46)
	*p*-value	<0.001	0.999	0.009
Partial 2	Block 1 (SE)	52.2 (3.05)	27.3 (0.96)	10.1 (2.61)
	Block 12 (SE)	63.5 (2.89)	26.0 (1.01)	23.0 (2.46)
	*p*-value	<0.001	0.999	<0.001

CB = Correct Behavior, DtC = Distance to Center, TD = Time Deviation. Means and *p*-values for practice Blocks 1 and 12; dependent variables CB, DtC, and TD.

**Figure 4:**
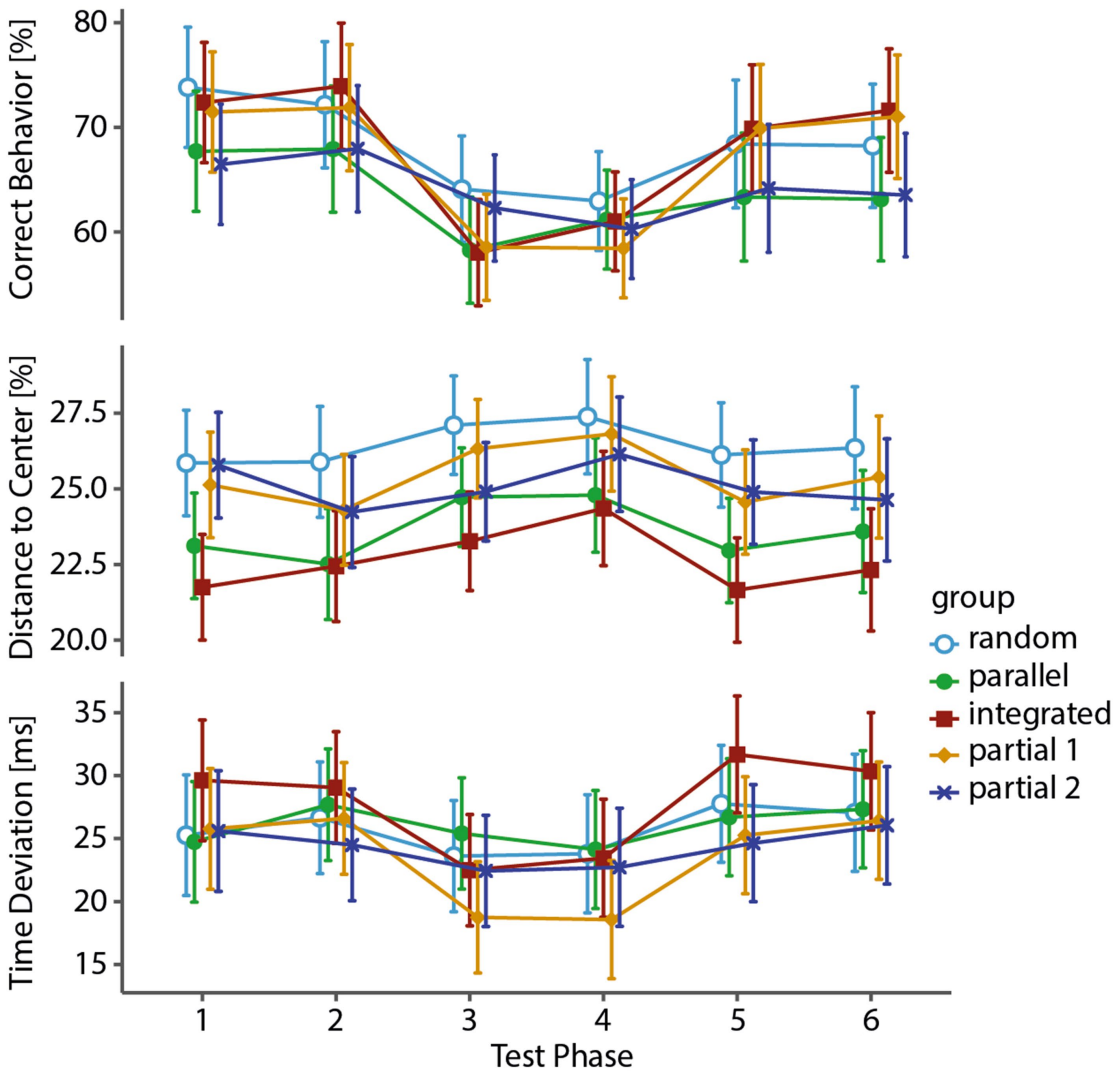
Performance changes during test phase. The graphs show the means (95% CI) of each test-phase block for each variable and group. The catch trial in blocks three and four lead to significant performance detriments, except for the Random, Partial 1, and Partial 2 group for DtC, and the Parallel group for TD.

We next analyzed the catch trial in the test phase (for overview, see Figure 4). We ensured the validity of calculating an ILS by first checking whether significant differences existed between familiar and random test phase blocks for all groups’ variables with either within-group dependent samples *t*-tests or Wilcoxon signed rank tests. This also ensures that performance increases can be attributed to learning of the underlying sequences and not just use-dependent learning. The Holm–Bonferroni method was used for *α* value correction. All catch trials for all groups registered significant differences, with the exception of DtC for the Random [*t* (19) = −1.77, *p* = 0.093, *α*adj = 0.025], Partial 1 [*t* (19) = 5.83, *p* = 0.048, *α*adj = 0.017] and Partial 2 [*t* (19) = −1.24, *p* = 0.230, *α*adj = 0.050] group, and TD for the Parallel [*t* (19) = 2.03, *p* = 0.057, *α*adj = 0.050] group.

**Figure 5:**
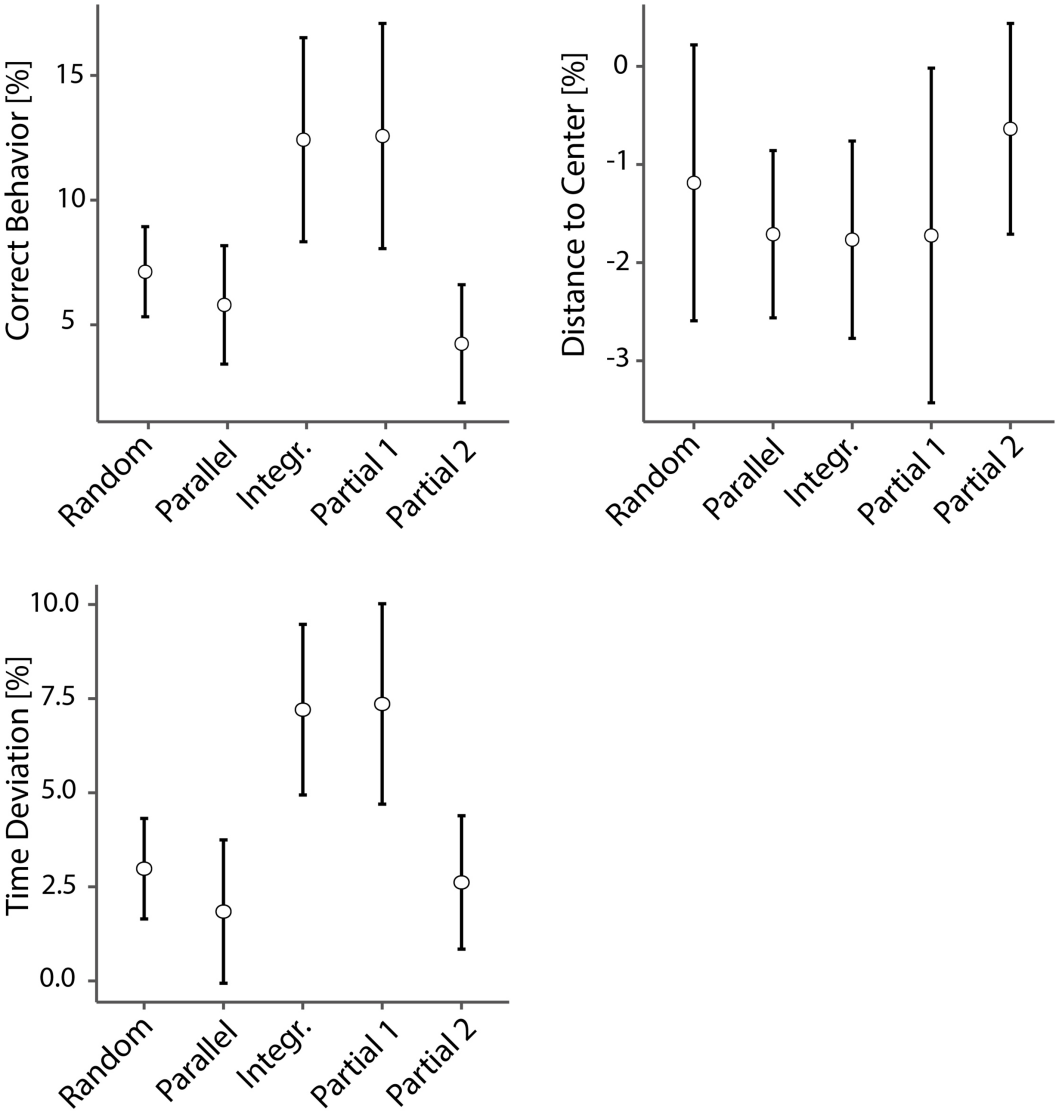
Implicit learning scores between groups. The respective implicit learning scores (mean 95% CI) for each group and each variable are shown. Fisher’s or Welch’s one-way between-groups ANOVAs with the dependent variable “implicit learning score” and the grouping variable “group” were used. Error bars represent standard errors. The Integrated and Partial 1 groups display the highest implicit learning scores for CB and TD, while the DtC scores are similar for all groups.

Overall, Time Deviation and Correct Behavior were lower and Distance to Center higher in the random blocks compared to the familiar ones, which allowed us to proceed with a comparison of the ILS between groups (see Figure 5) to compare their respective extent of implicit learning. To this end, we used either Fisher’s or Welch’s one-way between-groups ANOVAs with the dependent variable “implicit learning score” and the grouping variable “group” for CB, *F* (4, 46.4) = 5.13, *p* = 0.002, *η*^2^_part_ = 0.31, for DtC, *F* (4, 46.9) = 0.87, *p* = 0.491, *η*^2^_part_ = 0.07, and for TD, *F* (4, 95) = 7.48, *p* < 0.001, *η*^2^_part_ = 0.24. For DtC, no significant differences between the groups’ ILS could be shown at all. We followed up with *post-hoc* comparisons for CB and TD using the Games-Howell *post-hoc* test.

The analyses overall show several differences between the groups. For CB, the Integrated and Partial 1 group displayed a stronger drop in Correct Behavior with the former being significantly different to the Parallel (*p* = 0.047) and Partial 2 (*p* = 0.009) group, while the latter was approaching significance to the Parallel (*p* = 0.067) and showing clearly significant difference to the Partial 2 (*p* = 0.015) group. Clear changes in the participants’ rhythm with significant ILS differences between groups could be shown for TD. Again, the Integrated and Partial 1 group had benefitted from their normally stable sequences the most, deviating from their usual timing, with significant differences for both groups to the Random (*p* (int) = 0.023; *p* (p1) = 0.017), Parallel [*p* (int) = 0.002; *p* (p1) = 0.001], and Partial 2 (*p* (int) = 0.011; *p* (p1) = 0.007) group.

In summary, the catch-trial analysis and ILS showed that the random catch trial has distinctively affected all variables in all groups, with the exception of DtC for the Random group and TD for the Parallel group. As evidenced by group difference in ILS, differences in the degree of disruption between groups could mainly be shown for the variables Correct Behavior and Time Deviation. However, the changes in accuracy seemed to be rather uniform in all groups. As for the individual groups, most successful learning overall was displayed by the Integrated and Partial 1 group, while the other three groups were not significantly different from each other.

## 4 Discussion

This paper focused on the influence of task integration on implicit sequence learning in complex, continuous tasks, using VR as a research tool. We tested whether implicit sequence learning occurs in complex, continuous, predictable tasks and can thus improve performance more for groups with more suitable sequence structures. In regard to sequence structure in this context, we hypothesized that task integration should affect learners in a similar way as it has been shown to influence simple tasks and discrete, complex tasks (Beißel and Künzell, 2024; de Oliveira et al., 2017; Ewolds et al., 2021; Röttger et al., 2021; Schmidtke and Heuer, 1997). We further assessed whether task integration for these tasks can mitigate random perturbations and remain beneficial for motor learning. As we are assessing a continuous task, we sought alternative, yet comparable measures to the classic SRT task with an adapted VR SRT task and expected similar results.

In the interest of valid analyses and comparisons, we carefully assigned participants to their respective groups based on a pre-test to ensure that all groups would start out on a comparable performance level. We also interviewed participants at the end of the experiment to assess the level of explicit awareness they had reached about the repeating sequence used most of the time. The results of both assessments allow for the conclusion that differences in learning development can be attributed to the individual sequence structures used within each group and that for at least 90% of participants, no concrete explicit knowledge had been gained. As mentioned earlier, reliably drawing clear distinctions between implicit and explicit motor learning is a nigh impossible task and certainly not within the capability of a short interview to elucidate (Hadjiosif and Krakauer, 2021; Maresch et al., 2021; Moisello et al., 2009). Yet as a tendency towards implicit knowledge can be seen it allows for easier categorization and comparison of this study with earlier literature (e.g., Schmidtke and Heuer, 1997).

In order to show that implicit sequence learning is present in complex, continuous tasks, we analyzed changes in performance within each group by looking at beginning and end of the practice phase, and the impact of the random catch trial in the test phase. During the practice phase, distinct changes could be shown for all groups for CB, indicating a reduction of mistakes made and consequently at least partial learning of the underlying sequence.

Significant changes to TD could likewise be shown, but contrary to our expectations, an increase in TD occurred. Participants started out with very low Time Deviation, thus closely adhering to the experiment’s instructions, then progressively and uniformly increased their Time Deviation. This is not to be confused with an increase in RT, however, as the temporal-spatial point of reference used for measurement of TD was placed at a predefined point. The progressive shift in TD indicates that the predetermined movement rhythm or point of contact did not agree with the participants, and they adapted to a more individual rhythm instead. While we did not predict an increase in Time Deviation but a decrease, it can still serve as an indication for practice-induced changes in variability, especially as it was also affected by the catch trial in the test phase. Accuracy did apparently not change significantly within-group over the course of practice, although the DtC values decreased for all groups except the Random one.

Overall, these results show distinct evidence for motor learning during the practice phase, which can further be supported by the clearer results of the test phase. The within-group catch trial analyses show a clear decline in performance in the random blocks compared to the practiced sequence structure in most groups for CB and DtC, as well as a disruption of the familiar movement rhythm in TD. The only exceptions were DtC in the Random, Partial 1 and Partial 2 group, and TD in the Parallel group.

These results allow for several conclusions. First of all, we can assume that motor learning was successful overall as at least two variables within each group showed significant changes in response to the catch trial. The practice phase comparison further underlines this. This also indicates that not just bi-manual, integrated learning took place but also a certain degree of effector-specific learning, as both the Random and Parallel group were affected despite their group design making task integration impossible. This does not come as surprise, however, as previous studies have shown that while task integration may be a dominant and active influencing factor (Pelzer et al., 2022a; Pelzer et al., 2022b; Röttger et al., 2021; Zhao et al., 2020), alternative learning mechanisms are not suppressed and contribute to effector-specific learning for both simple (Bapi et al., 2000; Berner and Hoffmann, 2008, 2009; Verwey and Clegg, 2005; Verwey and Wright, 2004) and complex skills (Beißel and Künzell, 2024). The significant changes to TD are likewise an indicator of successful learning and the influence of alternative learning processes, as TD could have remained unaffected by the catch trial due to identical temporal stimulus presentation compared to familiar trials. Only the cube’s hit-direction prompts were changed in the random trials, which was nevertheless disruptive enough to also affect TD. And lastly, indications for the distinct influence of task integration in this experiment can be deduced from these within-group catch trial analyses as well, as the groups with non-significant catch trial differences in variables mentioned above, were those with the least ideal task integration conditions.

The primary measure for the influence of task integration, however, was the comparison of the groups’ success in acquiring the underlying sequences, represented by the implicit learning score. We expected the Integrated group to exhibit the highest scores, closely followed by the Partial 1 group and then the other groups. Based on our previous experiments, we did not expect the Random, Parallel, and Partial 2 group to significantly differ from each other, as they each served as a control group in their own way. The results indicated similar performance of these groups, as no significant ILS differences for any variable could be shown between them. The means revealed the Random and Partial 1 group to be largely similar, with Partial 2 displaying lower ILS values than both in almost all variables. In line with hypothesis 1, the Integrated group overall showed several differences towards the other three groups. In line with hypothesis 2, the Partial 1 group showed similar values as the Integrated group. The difference between the Integrated and the Partial 1 group to the other three groups was especially pronounced for the variable TD. In regard to DtC, the catch trial did apparently not influence accuracy at all, the reasons for which will be discussed later. For CB, the Integrated group was affected more than both the Parallel and Partial 2 group, while the Partial 1 group was only significantly different to Partial 2. In regard to our first hypothesis, these results show that task integration is indeed a beneficial factor in complex, continuous tasks. The two groups most suited for integrating their dual tasks, the Integrated and Partial 1 group, were overall more affected by the catch trial than the control groups and thus displayed greater learning success. This success can be explained by effective integration of the more favorable sequence structures used in their respective tasks. Regarding our second hypothesis, it also rather clearly shows that the Partial 1 group managed to mitigate the random interferences in their secondary task well enough to achieve a level of performance similar to the “ideal” task integration group. Task integration’s robustness against interferences seems to be limited, however, as the repeating covariations in the Partial 2 group did not lead to improved performance over the Random and Parallel group, which both could not integrate their respective sequences.

Yet not all variables painted an equally clear picture in this regard. Especially for CB, several differences in learning effects could be found between the Random and Parallel group compared to the Integrated and Partial 1 group which approached, but did not pass the threshold for statistical significance. One explanation for this might be found in the range of standard deviation in the respective groups, with far larger SDs being registered for the Integrated and Partial 1 group. This shows that within the Integrated and Partial 1 group, some participants were more affected by the catch trial than others, which might be traced back to individual learning and performance factors, such as an internal or external focus of attention, the ability to adapt to a new situation and reduce variability quickly, or simply motivational and attentional factors (Hossner and Zahno, 2022; Wulf and Lewthwaite, 2016; Wulf et al., 2010). With less to learn and still less to integrate in their task structures, the other groups should be less affected by the catch trial, leading to less variance. This tendency further supports the distinct results already gained from the ILS analyses and thus the arguments for the effects of task integration on complex, continuous tasks.

Overall, we can confirm our proposed hypotheses in this experiment: First, we have found results in our experiment that mirror those of similar studies on the topic of task integration. We show that dual-task interference in implicit motor learning can be mitigated if the sequence structure of the secondary task can be integrated with the primary task. This has been shown as a basic premise by Schmidtke and Heuer (1997) and reproduced in the context of complex motor tasks in our previous experiment (Beißel and Künzell, 2024). Similar results have been found by de Oliveira et al. (2017), who researched task integration in continuous tasks, using a tracking task. We show that the same holds true for complex, continuous, and predictable tasks. The design of our groups further supports the assumptions of Röttger et al. (2021) on the importance of predictability of across-task stimulus and/or response events between two tasks. Second, we have found that task integration processes can to some extent mitigate random perturbations in underlying sequence structures and remain beneficial by focusing on repeating covariations. This effect is nevertheless not universal, as an insufficient amount of covariations appears to remain undetected. However, as we base our claims on a novel approach towards measuring complex sequence learning, a critical assessment of the variables used in this experiment is certainly warranted and will be undertaken in the following.

As mentioned above, we have aimed for similar results as are already established in the field of task integration using alternative variables. Response times and error rates are frequently used variables in this field of study (Schmidtke and Heuer, 1997; Schwarb and Schumacher, 2012) and as such, we sought to mimic these as closely as possible with the variables “Time Deviation” and “Correct Behavior.” Measuring changes in accuracy was made possible by the variable “Distance to Center.” It was intended to measure deviation from the ideal standard, a hit placed in the very center of the target. This approach is similarly used for tracking experiments in the study of motor learning and task integration (de Oliveira et al., 2017; Ewolds et al., 2021). Accuracy measurements are not uncommon in the study of variability either and were thus adapted to our task (Kumar et al., 2017; Srinivasan et al., 2015).

Time Deviation was intended to measure the participants’ change in movement rhythm, as measured from a predetermined time point. A time point had to be set, due to the continuous nature of the task. Much as with classic RT measurements, we could compare TD at the beginning of the task with later points in time and could show significant changes. The changes in movement rhythm can be interpreted as successful learning in the form of an adaptation towards behavior fitting both the task and the individual’s preferences. The disruption of TD by the catch trial further strengthens this argument. We therefore consider TD a fitting variable for measuring temporal aspects of a continuous task.

Correct Behavior as a measure of error or success rate was likewise a rather fitting variable, yet as mentioned earlier, it has been established as such in earlier experiments. We could show a distinct increase in the percentage of successful hits during the practice phase, which was also disrupted in the test phase by the catch trial. It therefore comes as no surprise that CB can serve as a valid variable for continuous, complex tasks, also in VR.

Distance to Center as a measure of accuracy did not follow the pattern outlined for the previous variables. We expected an overall improvement in accuracy and an advantage for the groups more suited to task integration, as mental resources freed up by the integrated tasks could be used to focus on more precise hits. Yet while we could show significant improvement in accuracy over the practice phase and even some differences between groups, DtC did not change much in the test phase and was unaffected by the catch trial. We therefore must conclude that the improvement of accuracy reflects learning of the task set-up, but not necessarily of the underlying sequences, as accuracy did not change after reaching a certain level. It might therefore not be an ideal variable for assessing task integration or motor learning in this context but could be useful for solely measuring changes in variability over the course of adapting to a task.

We thus conclude that our variables have overall been adequate as a measure for motor learning in this VR task and, excluding DtC, are also satisfactory for assessing task integration. Alternative variables for a valid assessment of complex, continuous tasks in a head-mounted VR environment may naturally be possible, starting from directly recording movement data using the inbuild or additional sensors and continuing with task-specific, indirect variables. This broad accessibility towards designing an ideal environment perfectly suited for almost any experiment makes VR a useful tool (Dobrowolski et al., 2021; Levac et al., 2019; Olivier et al., 2021; Rizzo et al., 2019), especially for complex motor tasks. Yet researcher should ensure that stimuli appear within the participants’ field of vision to avoid search behavior (Arif et al., 2022), as well as be cautious about transfer of insights and skills to realistic tasks (Kim et al., 2019). More future research using VR should be encouraged and may help advance research on complex motor skill learning.

## 5 Conclusion

Sequential motor learning and task integration affect a great variety of motor skills in all aspects of life, making it a valuable research subject. We highlight the importance of re-assessing previously gained insights on simple motor tasks in the context of complex, continuous dual tasks, due to higher degrees of redundancy and variability in the latter. We find virtual reality technology to be a suitable and versatile tool in the assessment of complex motor tasks as it provides stable and highly controllable research environments. Consistent with previous research, our experiment on complex, continuous tasks shows that task integration represents a dominant influence on the learning of two simultaneously attempted tasks (Pelzer et al., 2022a; Röttger et al., 2021) and can have mitigating effects on dual-task costs if covariations between the tasks can successfully be identified and integrated (de Oliveira et al., 2017; Röttger et al., 2021). Conversely, task acquisition is disrupted through task confusion if covariations do not exist or are too inconsistent to be integrated (Hazeltine and Schumacher, 2016; Röttger et al., 2021). Furthermore, minor inconsistencies in one motor task can be compensated to a certain degree and still lead to successful overall sequence learning. This study assesses task integration in the context of sequence learning for complex, continuous dual tasks. While it generally shows results consistent with previous research on simple tasks, more extensive research on complex tasks is needed to allow for an eventual transfer of knowledge out of the laboratory and into the field.

## Data Availability

The datasets presented in this study can be found in online repositories. The names of the repository/repositories and accession number(s) can be found at: https://rb.gy/wj57q9 to be found at Open Science Framework (OSF).

## Footnotes

^1^ https://rb.gy/wj57q9
